# Robotic vs. laparoscopic liver surgery: a single-center analysis of 600 consecutive patients in 6 years

**DOI:** 10.1007/s00464-021-08770-x

**Published:** 2022-05-31

**Authors:** Moritz Schmelzle, Linda Feldbrügge, Santiago Andres Ortiz Galindo, Simon Moosburner, Anika Kästner, Felix Krenzien, Christian Benzing, Matthias Biebl, Robert Öllinger, Thomas Malinka, Wenzel Schöning, Johann Pratschke

**Affiliations:** 1 Department of Surgery, Charité – Universitätsmedizin Berlin, Corporate Member of Freie Universität Berlin and Humboldt-Universität zu Berlin, Campus Charité Mitte and Campus Virchow-Klinikum, Augustenburger Platz 1, 13353 Berlin, Germany; 2 Berlin Institute of Health at Charité – Universitätsmedizin Berlin, Charitéplatz 1, 10117 Berlin, Germany

**Keywords:** Robotic liver surgery, Laparoscopic liver surgery, Surgical robot, Robotic surgery

## Abstract

**Background:**

While laparoscopic liver surgery has become a standard procedure, experience with robotic liver surgery is still limited. The aim of this prospective study was to evaluate safety and feasibility of robotic liver surgery and compare outcomes with conventional laparoscopy.

**Methods:**

We here report the results of a single-center, prospective, post-marketing observational study (DRKS00017229) investigating the safety and feasibility of robotic liver surgery. Baseline characteristics, surgical complexity (using the IWATE score), and postoperative outcomes were then compared to laparoscopic liver resections performed at our center between January 2015 and December 2020. A propensity score-based matching (PSM) was applied to control for selection bias.

**Results:**

One hundred twenty nine robotic liver resections were performed using the da Vinci Xi surgical system (Intuitive) in this prospective study and were compared to 471 consecutive laparoscopic liver resections. After PSM, both groups comprised 129 cases with similar baseline characteristics and surgical complexity. There were no significant differences in intraoperative variables, such as need for red blood cell transfusion, duration of surgery, or conversion to open surgery. Postoperative complications were comparable after robotic and laparoscopic surgery (Clavien–Dindo ≥ 3a: 23% vs. 19%, *p* = 0.625); however, there were more bile leakages grade B–C in the robotic group (17% vs. 7%, *p* = 0.006). Length of stay and oncological short-term outcomes were comparable.

**Conclusions:**

We propose robotic liver resection as a safe and feasible alternative to established laparoscopic techniques. The object of future studies must be to define interventions where robotic techniques are superior to conventional laparoscopy.

The advantages of the minimally invasive approach to liver resection have been demonstrated in numerous studies [1–3]. Compared to the conventional open approach, laparoscopic liver surgery is associated with a lower complication rate, less postoperative pain, shorter hospital stay and a higher quality of life [2, 4]. According to a recent randomized trial, these benefits can be achieved without compromising oncologic long-term results [5]. Laparoscopy has therefore become the standard technique at our center and many others worldwide, with conventional open liver surgery being reserved for extended indications, e.g., resections with vascular or biliary reconstruction.

By enabling 3-D visualization and permitting a greater range of motion, robotic liver surgery has been reported to improve surgeon ergonomics, surgical accuracy and to reduce surgeon fatigue [6, 7]. In this respect, the robotic technique might be particularly suitable for highly complex liver resections and could offer potential advantages over laparoscopy. Even though the first case series on robotic liver surgery were published back in the early 2000s, the robotic technique has surprisingly not spread widely [6, 7]. This is certainly associated, at least in part, with the high initial costs of robotic surgical systems and the high running costs [8, 9].

However, interest in implementing robotic techniques in liver surgery has increased dramatically in the last few years. Since recent series have produced promising results, it is now time to safely accompany the development of robotic liver surgery within the framework of prospective studies [10–13]. Suggested benefits need to be verified and validated, especially with regard to an improved case selection and potential technical limitations that may require further refinement and innovation for robotic surgical systems. In this study, we, therefore, present our data from a prospective single-center observational study investigating the safety and feasibility of robotic liver surgery and compare results to established laparoscopic techniques.

## Materials and methods

### Study design and data collection

We here report the results of a single-center, prospective, post-marketing observational study (DRKS00017229) with the objective of investigating clinical outcomes of robotic liver surgery using the da Vinci Xi surgical system (Intuitive, Sunnyvale, CA, USA). All patients who underwent robotic liver resection at the Department of Surgery, Campus Charité Mitte and Campus Virchow-Klinikum, Charité-Universitätsmedizin Berlin, Germany, between July 31^st^, 2017 and December 15th, 2020, were included. Baseline characteristics, intraoperative technical details including dissection devices, duration of surgery and console time, as well as postoperative complications were prospectively recorded and analyzed.

All included patients gave informed consent to the collection of their personal and medical data and its use for research purposes. All data were collected, stored, and processed according to the General Data Protection Regulation and local data protection laws. The study was conducted in accord with the ethical standards of the Helsinki Declaration of 1975. The Charité Institutional Review Board (IRB) approved of the study (EA4/084/17).

In a second step, baseline characteristics, surgical complexity and postoperative outcomes of all consecutive laparoscopic liver resections performed at our center between January 2015 and December 2020 were retrospectively analyzed and compared to the robotic approach. Parts of these data have been published previously [14, 15]. The clinical data were handled according to the same ethical and data protection guidelines after prior IRB approval (EA2/006/16). A propensity score-based matching (PSM) was performed to control for selection bias.

### Patient selection and perioperative management

Liver resections were preferentially scheduled for minimally invasive surgery in the absence of specific reasons for open resection. These could be patient’s preference or technical issues, e.g., the need for vascular or biliary reconstruction. Before the surgical robot was installed in our program and this prospective observational study was started, all minimally invasive liver resections were performed laparoscopically. After obtaining initial experience with minor resections, the robotic approach was increasingly applied for all extents of resection. The decision to use laparoscopic or robotic approach was not based on defined selection criteria. Preoperative staging included helical computed tomography (CT) scanning of the chest, abdomen and pelvis, which was individually supplemented or replaced by magnetic resonance imaging (MRI) of the liver. All cases of suspected primary or secondary malignant tumors were discussed at one of our weekly multidisciplinary tumor boards. Before extended resection, liver augmentation was induced as individually indicated.

### Surgical techniques

Laparoscopic liver resection was performed as reported previously [14–16]. Port strategies included multi-incisional laparoscopic surgery (MILS) and in the early phase single incision laparoscopic surgery (SILS) and hand-assisted laparoscopic surgery (HALS).

For robotic surgery, the da Vinci Xi surgical system (Intuitive, Sunnyvale, CA, USA) was used. Patients were positioned in reverse Trendelenburg position. Four 8 mm robotic trocars were supplemented by two 12 mm (± one 5 mm) assist trocars. A standard operating procedure for the positioning of the patient, port placement, setting of the robotic arms and differences to the laparoscopic approach has been described by our group in detail elsewhere [17].

In both laparoscopic and robotic surgery, ultrasound was routinely performed intraoperatively to confirm the exact tumor location, boarders, proximity to vascular and biliary structures and to rule out further intrahepatic lesions. In preparation for an extracorporeal intermittent Pringle maneuver (IPM), the hepatoduodenal ligament was isolated and threaded, and 250 mg methylprednisolone was administered intravenously prior to parenchymal resection. IPM was applied according to the surgeon's individual decision with 15-min intervals interrupted by 5 min of reperfusion each. In case of iCC, hilar lymph node dissection and intraoperative frozen section analysis were performed routinely, irrespective of the access technique.

In laparoscopy, superficial parenchymal dissection was performed by using energy shears (Harmonic ACE, Ethicon, Inc., Somerville, NJ, USA and THUNDERBEAT, Olympus K.K., Tokyo, Japan). Options for deeper parenchymal dissection included laparoscopic cavitron ultrasonic surgical aspirator (CUSA, Valleylab Boulder, CO, USA), waterjet (ERBEJET, Elektromedizin GmbH, Tübingen, Germany) and in some cases during the learning curve also vascular staplers (Echelon, Ethicon, Somerville, NJ, USA). As these options are not available for use with the da Vinci Xi surgical system, parenchymal transection was performed in a modified clamp crush technique using Harmonic ACE curved shears. Both in laparoscopic and robotic liver surgery, large vessels were either clipped or transected using staplers.

Routinely, a drain was inserted in the abdomen, which was removed on the second postoperative day in case of normal fluids. Patients with liver cirrhosis and after major resections were referred to the intensive care unit (ICU) postoperatively, while individual decisions were made for patients after minor resections.

### Clinical parameters and classifications

The preoperative general status of our patients was assessed using the American Society for Anesthesiologists physical status classification (ASA). The underlying pathology of the resected tumor was determined by the staff pathologist in all cases. In case of a malignant tumor, TNM status and resection margin (R status) were specified. Presence of liver fibrosis was also described by histopathology and graded according to Desmet et al. [18]. Furthermore, clinical stage of liver cirrhosis was classified according to the Child–Pugh score [19].

The assessment of surgical complexity was based on the IWATE criteria [20] and the extent of resection (major resection vs. minor resection). Major resection was defined as resection of three segments or more [21]. Furthermore, tumor size (< 3 cm vs. ≥ 3 cm) and tumor location were reported as categorized by the IWATE classification system. Here, liver segments are grouped by the difficulty of access and awarded between one and five points within the scoring system [22].

Postoperative complications within 90 days after surgery were classified according to Clavien–Dindo [23]. Bile leakage was graded according to the International Study Group of Liver Surgery (ISGLS) definition [24]. Further intra- and postoperative outcome parameters included duration of surgery, intraoperative need for transfusion of red blood cells (RBCs), length of stay in the ICU and length of stay in the hospital (LOS). Serum levels of bilirubin, alanine aminotransferase (ALT), aspartate aminotransferase (AST), bilirubin and the international normalized ratio (INR) are reported as preoperatively, on the first postoperative day and the last value before discharge.

### Statistical analysis

IBM SPSS Statistics version 25 (IBM Corp., Armonk, NY, USA) and R version 4.03 (R Foundation for Statistical Computing, Vienna, Austria) were used for data analysis. Categorical data were analyzed using Pearson’s *χ*^2^ test and reported as frequencies and percentages. Continuous data were tested for normality using the Shapiro–Wilk test and analyzed accordingly by Mann–Whitney *U* test or Student’s *t* test. Nonparametric data are presented as median and range (minimum–maximum), parametric data are reported as mean and standard deviation. A *p* value < 0.05 was considered statistically significant. The propensity score matching was performed with R using the package “Matchit”, with nearest neighbor one to one matching, without replacement and a caliper of 0.2. The following variables were included in the propensity score model: tumor size ≥ 3 cm, tumor location according to IWATE score, extent of resection (major vs. minor), presence of liver cirrhosis, body mass index and severe systemic disease (ASA ≥ 3).

## Results

### Prospective analysis of robotic liver resections

From the first robotic liver resection in April 2018 until the end of our study in December 2020, 129 liver resections were performed using the da Vinci Xi surgical system and included in our prospective observational study. Most patients (81%) had malignant primary or secondary liver tumors. The most common tumor entity was colorectal liver metastasis (CRLM, 28%), followed by hepatocellular carcinoma (HCC, 23%) and intrahepatic cholangiocarcinoma (iCC, 12%). All baseline characteristics are described in Table 1.

**Table 1:** Baseline characteristics before and after propensity score matching

	Pre-PSM					Post-PSM				
	Laparoscopic		Robotic		*p* value	Laparoscopic		Robotic		*p* value
	*n* = 471		*n* = 129			*n* = 129		*n* = 129		
Age (years)	62	(19–88)	64	(22–85)	0.836	62	(19–86)	64	(22–85)	0.802
Sex (female)	211	(45%)	63	(49%)	0.426	59	(46%)	63	(49%)	0.708
BMI (kg/m^2^)	26	(17–44)	25	(17–40)	0.201	25	(17–44)	25	(17–40)	0.613
ASA										
< 3	246	(52%)	65	(50%)	0.766	58	(45%)	65	(50%)	0.455
≥ 3	225	(48%)	64	(50%)		71	(55%)	64	(50%)	
Liver cirrhosis										
No cirrhosis	366	(78%)	112	(87%)	0.052	112	(87%)	112	(87%)	0.597
Child–Pugh A	99	(21%)	17	(13%)		16	(12%)	17	(13%)	
Child–Pugh B	5	(1%)	0			1	(1%)	0		
Pathology										
HCC	121	(26%)	30	(23%)	0.057	26	(20%)	30	(23%)	0.359
CRLM	166	(35%)	36	(28%)		51	(40%)	36	(28%)	
iCC	31	(6%)	16	(12%)		12	(9%)	16	(12%)	
Other-malignant	56	(12%)	23	(18%)		17	(13%)	23	(18%)	
Benign	97	(21%)	24	(19%)		23	(18%)	24	(19%)	

*Pre-PSM* all patients before propensity score matching, *Post-PSM* after propensity score matching, *BMI* body mass index, *ASA* American Society for Anesthesiologists physical status classification, *HCC* hepatocellular carcinoma, *CRLM* colorectal liver metastasis, *iCC* intrahepatic cholangiocarcinoma

Roughly half (47%) of the robotic liver resections were major resections (Table 2). According to the IWATE classification, the median surgical difficulty score was nine, corresponding to “advanced” difficulty, with 36% of cases classified as > 10, corresponding to “expert” level [20].

**Table 2:** Complexity of surgery before and after propensity score matching

		Pre-PSM					Post-PSM				
		Laparoscopic		Robotic		*p* value	Laparoscopic		Robotic		*p* value
		*n* = 471		*n* = 129			*n* = 129		*n* = 129		
Extent of resection											
Minor		347	(74%)	68	(53%)	< *0.001*	68	(53%)	68	(53%)	1.000
Major	Right	87	(18%)	40	(31%)		40	(31%)	40	(31%)	
	Left	37	(8%)	21	(16%)		21	(16%)	21	(16%)	
IWATE score		7	(1–11)	9	(1–12)	< *0.001*	8	(2–11)	9	(1–12)	0.337
Tumor ≥ 3 cm		248	(53%)	89	(69%)	*0.001*	84	(65%)	89	(69%)	0.596
Location score (IWATE)											
1	Segment III	22	(5%)	3	(2%)	*0.001*	4	(3%)	3	(2%)	0.257
2	Segment II/VI	99	(21%)	12	(9%)		19	(15%)	12	(9%)	
3	Segment IVb/V	67	(14%)	24	(19%)		16	(12%)	24	(19%)	
4	Segment I/IVa	45	(10%)	25	(19%)		17	(13%)	25	(19%)	
5	Segment VII/VIII	238	(50%)	65	(50%)		51	(57%)	65	(50%)	

*p* values <0.05 are indicates in italic

*Pre-PSM* all patients before propensity score matching. *Post-PSM* after propensity score matching

The median duration of surgery was 260 min (83–568), with 167 min median console time (47–384). In 56% of the cases, IPM was applied, with a median total duration of 16 min (3–68) (Table 3). For 37%, a vascular stapler was used for transection of large vessels. In seven cases (5%), the surgery was converted to open or laparoscopic technique. Six patients received RBC intraoperatively (5%). Seventy-six percent of cases with malignant tumors were resected with histological declaration of tumor-free margin (R0).

**Table 3:** Intraoperative and postoperative outcome criteria before and after propensity score matching

	Pre-PSM					Post-PSM				
	Laparoscopic		Robotic		*p* value	Laparoscopic		Robotic		*p* value
	*n* = 471		*n* = 129			*n* = 129		*n* = 129		
Duration of surgery (min)	244	(45–758)	260	(83–568)	0.135	270	(57–580)	260	(83–568)	0.613
Port strategy										
SILS	21	(4%)				3	(2%)			
HALS	106	(23%)				35	(27%)			
Laparoscopic MILS	339	(73%)				91	(71%)			
Robotic MILS			129	(100%)				129	(100%)	
Use of IPM	219	(47%)	72	(56%)	0.084	70	(54%)	72	(56%)	0.900
Total duration of IPM	25	(5–113)	16	(3–68)	*0.003*	26	(5–113)	16	(3–68)	0.050
Conversion	16	(3%)	7	(5%)	0.302	6	(5%)	7	(5%)	1.000
Intraoperative RBC transfusion	35	(8%)	6	(5%)	0.327	11	(9%)	6	(5%)	0.315
LOS: ICU (days)	1	(0–43)	1	(0–81)	0.125	1	(0–24)	1	(0–81)	0.405
LOS: hospital (days)	8	(3–59)	8	(4–94)	0.471	8	(3–52)	8	(4–94)	0.816
R0 resection	302	(81%)	81	(76%)	0.118	87	(82%)	81	(76%)	0.407
90-day complications^a^										
None	296	(63%)	80	(62%)		83	(64%)	80	(62%)	
1–2	103	(22%)	19	(15%)	*0.042*	22	(17%)	19	(15%)	0.625
3–5	72	(15%)	30	(23%)		24	(19%)	30	(23%)	
Mortality	5	(1%)	1	(1%)	1.000	2	(2%)	1	(1%)	1.000
Bile leakage	49	(10%)	22	(17%)	*0.045*	15	(12%)	22	(17%)	0.286
Bile leakage: Grade^b^										
None	422	(90%)	107	(83%)	< *0.001*	114	(88%)	107	(83%)	*0.006*
A	24	(5%)	0			6	(5%)	0		
B	21	(5%)	20	(15%)		7	(5%)	20	(15%)	
C	4	(1%)	2	(2%)		2	(2%)	2	(2%)	

*p* values <0.05 are indicates in italic

*Pre-PSM* all patients before propensity score matching, *Post-PSM* after propensity score matching, *SILS* single incision laparoscopic surgery, *HALS* hand-assisted laparoscopic surgery, *MILS* multi-incisional laparoscopic surgery, *RBC* red blood cells, *ICU* intensive care unit, *LOS* length of stay, *IPM* Intermittent Pringle Maneuver

^a^According to Clavien–Dindo classification [23]

^b^According to the International Study Group of Liver Surgery definition [24]

After robotic liver resections, patients spent a median of one night at the ICU, and were discharged from hospital after a median of 8 days. Ninety-day postoperative overall morbidity was 38%, with severe complications (Clavien–Dindo ≥ 3) occurring in 23% of all cases. The most frequent type of complication was bile leakage, which was detected in 22 cases (17%, Table 3). Two of these cases required surgery, classifying as grade C bile leakage, while the remaining 20 cases were grade B. One patient (1%) died after robotic liver resection. This cirrhotic patient, who also had a pre-existing pulmonary disease, suffered a bile leakage grade C after right hepatectomy and subsequently developed pneumonia and multiorgan failure. All severe complications (Clavien–Dindo ≥ 3) are listed in Table 4.

**Table 4:** Severe complications according to Clavien–Dindo classification (≥ 3)

	Post-PSM			
	Laparoscopic		Robotic	
	n = 24		n = 30	
Bile leakage	8	(34%)	17	(57%)
Abdominal fluid collection	5	(21%)	6	(20%)
Pleural effusion	3	(13%)		
Pneumothorax			1	(3%)
Intestinal perforation	1	(4%)	1	(3%)
Fascial dehiscence	2	(8%)	1	(3%)
Hematoma	2	(8%)	1	(3%)
Acute pancreatitis			1	(3%)
Wound infection	1	(4%)	1	(3%)
Multi-organ failure, death	2	(8%)	1	(3%)

### Robotic vs. laparoscopic liver surgery: baseline characteristics

Between January 2015 and December 2020, 471 laparoscopic liver resections were performed at our center. All clinical data were retrospectively analyzed and compared with patients who underwent robotic liver resection. A PSM was performed to control for selection bias and potential confounding factors, including denominators of surgical difficulty (tumor size, tumor location score and extent of resection) and baseline characteristics (BMI, ASA score and presence of liver cirrhosis). After matching, 129 cases were included in each group.

Baseline characteristics were similar in patients who underwent laparoscopic and those who were scheduled for robotic surgery (Table 1). Eighty percent of all patients were operated for malignant and 20% for benign liver diseases, with no differences between both groups. Before matching, there was a trend toward more CRLM in the laparoscopic and more iCC in the robotic group, with liver cirrhosis less commonly seen in the robotic group; however, those differences were short of statistical significance. After PSM, these parameters were similar in both groups.

### Robotic vs. laparoscopic liver surgery: surgical complexity

When comparing all minimally invasive liver resections before matching, those in the robotic group were considerably more extensive and complex when compared to the laparoscopic group (Table 2). Forty-seven percent of robotic liver resections were major resections, compared to 26% in the laparoscopic group (*p* < 0.001). The median IWATE score as a measurement for surgical complexity was nine for robotic and seven for laparoscopic resections (*p* < 0.001). The percentage of large tumors (defined as ≥ 3 cm) was significantly higher in the robotic group than in the laparoscopic group (69% vs. 53%, *p* = 0.001). Regarding the tumor location score of the IWATE criteria, robotic liver surgeries were more likely performed for tumors in the segments classified as more complex (3–4 points: segments I, IV or V), while laparoscopically resected tumors were more often in the lower graded segments (1–2 points: segments II, III or VI).

### Robotic vs. laparoscopic liver surgery: Intraoperative outcome

After PSM, duration of surgery was comparable between both surgical approaches (robotic 260 vs. laparoscopic 270 min, n.s.). Intraoperative need for RBC transfusion was also similar in both groups (5% robotic vs. 9% laparoscopic, n.s.). Rates of conversion to open (or laparoscopic, in case of robotic) surgery were 5% during both surgical approaches. The resection status in malignant tumors was similar in both groups with 76% R0 status in the robotic and 82% in the laparoscopic group. Intraoperative and postoperative outcome criteria after robotic vs. laparoscopic liver resection both unmatched and after PSM are detailed in Table 3.

### Robotic vs. laparoscopic liver surgery: postoperative outcome

After surgery, median LOS was similar in both groups with one night at the ICU and 8 days in the hospital. After matching for surgical complexity, both overall morbidity and severity of complications were comparable in the robotic and laparoscopic groups.

The overall incidence of bile leakage was similar after robotic and laparoscopic liver resections after PSM (12% vs. 17%, n.s.). However, when considering the severity according to ISGLS criteria, cases of bile leakage after robotic surgery were graded significantly higher than in the laparoscopic group. After PSM, this difference remained significant with all cases in the robotic group graded B or C compared to 9 out of 15 (60%) in the laparoscopic group (*p* = 0.006), representing the only notable distinction in measured outcome criteria between the robotic and the laparoscopic surgical approaches. When classifying severe bile leakage (B–C) according to localization, 77% of bile leakage after robotic surgery were localized in the parenchymal dissection plane vs. 67% after laparoscopic resection (n.s.).

The causes and frequencies of severe complications (Clavien–Dindo ≥ 3a) of both groups after PSM are detailed in Table 4.

**Fig. 1:**
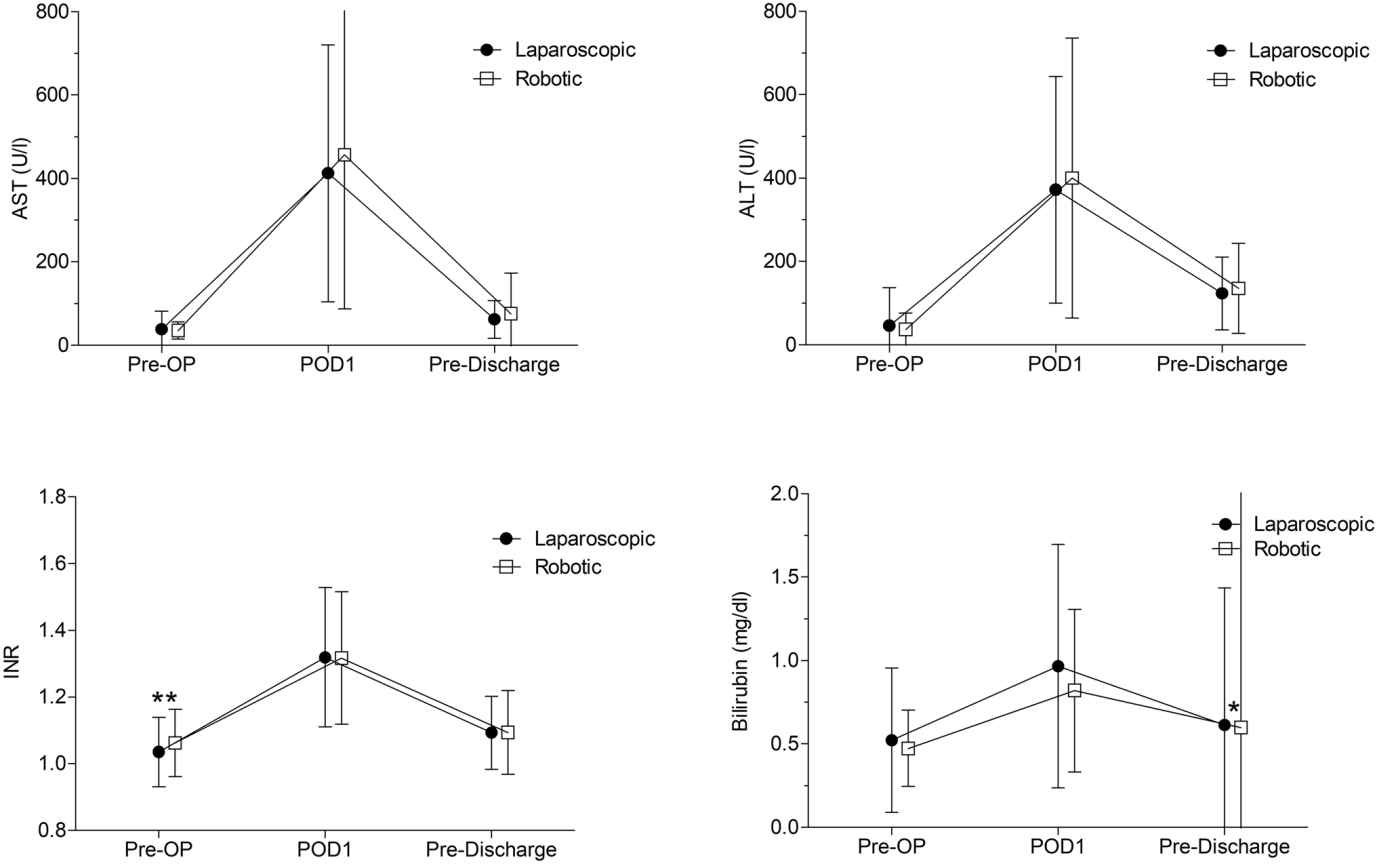
Serum levels of alanine aminotransferase (ALT), aspartate aminotransferase (AST), and bilirubin and international normalized ratio (INR) preoperatively (Pre-OP), on the first postoperative day (POD1), and before discharge from the hospital (Pre-Discharge). *U*/*l* units per liter, *mg*/*dl* milligram per deciliter. *p < 0.05, **p < 0.01

There were similar levels of serum markers of liver injury and liver function (ALT, AST, INR, bilirubin) preoperatively, with a slightly higher INR in the robotic group (1.06 robotic vs. 1.04 laparoscopic, *p* = 0.006, Fig. 1). On the first postoperative day there was no difference between groups. AST, ALT or INR also did not show any difference in the last test before release from the hospital. However, serum bilirubin was slightly, but statistically significantly lower in the robotic group at this time point, when compared to the laparoscopic group (0.60 mg/dl vs. 0.61 mg/dl, *p* = 0.034).

## Discussion

We here report our center’s experience in robotic liver surgery with the da Vinci Xi system as investigated in a prospective study. In synopsis with other series, our data indicate that robotic liver surgery is technically feasible and that implementation at a center for minimally invasive hepatopancreatobiliary surgery is safely possible within a short period of time [9, 25, 26]. The high average complexity of the interventions with around fifty percent major liver resections suggests that robotic assistance may be particularly advantageous in technically demanding procedures. Compared to our retrospective data from laparoscopic liver surgery, there are no clinically relevant differences in terms of overall perioperative morbidity or short-term oncologic outcomes.

The implementation of robotic liver surgery built on our experience of 471 laparoscopic liver resections since 2015. Profound technical and strategic experience in laparoscopic liver surgery was helpful in safely implementing the robotic program at our center. Well-known advantages of robotic assistance, such as 3-D visualization or instrument triangulation, were another important factor to safely switch from laparoscopic to robotic liver surgery and quickly led to a preference of the da Vinci Xi system especially for large tumors and complex resections. Accordingly, both the percentage of major liver resections and surgical complexity were higher in the robotic than in the laparoscopic group before matching.

Other groups also reported the perceived benefits of the robotic technique; however, they do not appear to translate into improved short-term outcomes compared to established minimally invasive techniques. Both the intraoperative and postoperative metrics are in the range of what previous studies have described for robotic liver surgery [25, 27]. In accordance, there were no significant perioperative improvements to our data obtained in laparoscopic liver surgery. Intraoperative characteristics such as duration of surgery, need for RBC transfusion or conversion to open surgery were comparable between both groups. Further, no significant differences to laparoscopic liver surgery were found with regard to postoperative complication rates, hospital stay, and short-term oncologic data.

While some other groups report similarly increased risk of bile leakage with robotic surgery [27, 28], a recent meta-analysis comprising 26 non-randomized studies found no significant difference to laparoscopic surgery [29]. In our study, bile leakage was more likely to be grade B or C after robotic liver surgery when compared to the laparoscopic group. This is an important finding, as these complications might require interventional or surgical treatment. It is conceivable that the transection technique in robotic surgery is causative, which is limited to a modified clamp crush technique due to a lack of alternatives. In this respect, there is an urgent need for the development of compatible dissection techniques in future robot generations, e.g., CUSA and waterjet. The higher median surgical complexity with higher frequency of iCC in the robotic group could also add to the higher rate of clinically relevant bile leakage. Another potential contributing factor may be the learning curve, as the era of robotic surgery within our study period was considerably shorter than of laparoscopy.

Our study has obvious limitations. Data in robotic surgery were collected prospectively as part of a study. Nevertheless, the comparison to laparoscopic surgery was only possible on the basis of retrospective data with inherent higher risk of confounding factors. Further, the results of the robotic cases must be interpreted with caution because they were collected within the learning curve, which is also true for the early laparoscopic cases. Although we did not establish clearly defined indications for using the robotic or the laparoscopic approach, a selection bias to either minimally invasive approach must be considered for the era of robotic surgery, which we tried to minimize by propensity score matching. Consequently, a final comparison of the techniques would require a prospective randomized study after completion of the learning curve which will also have to address the issue of bile leakage.

In conclusion, robotic liver resection can be safely established within a manageable period. This seems to be due to a shorter learning curve for robotic when compared to laparoscopic surgery [30, 31]. Whether the robotic technique is generally superior to the laparoscopic technique cannot be concluded based on our data. There are even some disadvantages, such as the currently limited choice of dissection techniques. However, there is the subjective impression that the strengths come into play especially in complex resections. In this respect, we are convinced that the technology will be a valuable addition to established minimally invasive techniques.
